# Diagnostic Documentation and Tumour Marker Availability Across Clinical Pathways in Real-World Gastric Cancer

**DOI:** 10.3390/diagnostics16071002

**Published:** 2026-03-26

**Authors:** Alexandru-Marian Vieru, Virginia-Maria Rădulescu, Maria-Lorena Mustață, Emil Trașcă, Sergiu-Marian Cazacu, Petrică Popa, Tudorel Ciurea

**Affiliations:** 1 Doctoral School, University of Medicine and Pharmacy of Craiova, 200349 Craiova, Romania; 2 Faculty of Medicine, University of Medicine and Pharmacy of Craiova, 200349 Craiova, Romania

**Keywords:** gastric cancer, TNM staging, incomplete staging, real-world evidence, tumour markers, CEA, CA19-9, diagnostic pathways, metastatic disease, observational study

## Abstract

**Background/Objectives**: In real-world gastric cancer cohorts, incomplete TNM staging and heterogeneous biomarker testing may result from structural characteristics of diagnostic pathways rather than random data loss. This study aimed to evaluate staging completeness and tumour marker availability as pathway-linked phenomena and to examine their associations with metastatic presentation and treatment allocation at diagnosis. **Methods**: This retrospective observational study included consecutive patients with histologically confirmed gastric carcinoma or adenocarcinoma diagnosed between 2018 and 2021 at a tertiary referral centre. Incomplete staging was defined a priori as the presence of Tx and/or Nx and/or Mx. The primary analytic endpoint was incomplete staging within the subset of patients with defined M status. Secondary analyses evaluated the availability of both CEA and CA19-9. Univariable associations were assessed using Pearson’s χ^2^, and multivariable logistic regression models estimated adjusted odds ratios (ORs) with 95% confidence intervals (CIs). Predefined pathway-oriented endpoints were analysed using multivariable logistic regression models adjusted for key clinical and diagnostic variables. **Results**: Among 375 patients, incomplete staging occurred frequently and was strongly associated with metastatic disease (M1) and non-surgical management. In multivariable analysis, metastatic presentation remained independently associated with incomplete staging, whereas surgical management and explicit documentation of disease extension were inversely associated. Concurrent availability of CEA and CA19-9 was concentrated within non-surgical and metastatic pathways and was independently associated with documented disease extension. These findings suggest that both staging completeness and tumour marker testing are determined by pathway-specific structures rather than random processes. **Conclusions**: In real-world gastric cancer care, incomplete TNM staging and tumour marker availability function as measurable features of diagnostic architecture rather than random data limitations. By modelling documentation completeness and testing availability as pathway-dependent phenomena, this study provides a pragmatic framework for improving transparency and interpretability in observational oncology research.

## 1. Introduction

Gastric cancer continues to pose a significant global health challenge, remaining among the leading causes of cancer-related mortality worldwide despite gradual declines in incidence in certain regions [1]. Projections indicate that, in absolute terms, the burden will remain considerable in the coming decades, primarily due to population ageing and ongoing exposure to risk factors, especially in areas with uneven access to diagnostic and therapeutic resources [2]. Consequently, the quality of care now depends not only on the availability of guideline-recommended treatments but also on the consistency and completeness of the diagnostic pathway preceding treatment allocation.

Accurate staging is fundamental to contemporary gastric cancer management. International guidelines emphasise TNM-based stratification to inform decisions regarding surgical eligibility, perioperative systemic therapy, and palliative interventions, with distant metastatic status (M1) serving as a critical determinant in clinical decision-making [3,4]. The TNM system aims to standardise assessment of disease extent and facilitate comparability across patient cohorts; however, its implementation in clinical practice is often inconsistent. The 8th edition of the staging system introduced refinements relevant to gastric cancer and underscored the importance of structured staging documentation. Nevertheless, real-world datasets frequently exhibit partial or missing TNM elements (such as Tx, Nx, and/or Mx) due to variable diagnostic work-up, limited imaging availability, fragmented referral processes, or incomplete reporting [5].

Notably, missing data in staging are often non-random. Large registry-based analyses across cancer types have demonstrated that missing stage-related information is common and can be systematically associated with patient characteristics, disease severity, and outcomes, potentially biasing inference if not explicitly addressed and reported [6]. In gastric cancer, incomplete staging complicates the interpretation of treatment patterns and may obscure clinically relevant phenotypes, particularly when patients are rapidly triaged to supportive care, referred late, or managed outside standardised multidisciplinary pathways. From a real-world evidence perspective, it is essential to make the diagnostic pathway both visible and measurable; incomplete staging should be recognised as a clinically relevant aspect of the care process rather than a technical inconvenience.

Alongside imaging- and pathology-based staging, serum tumour markers—most notably carcinoembryonic antigen (CEA) and carbohydrate antigen 19-9 (CA19-9)—are widely utilised in clinical practice. Although these markers are insufficient for diagnosis and are not recommended as standalone decision tools, they can provide additional biological context regarding tumour burden and risk, and are frequently incorporated into follow-up protocols in many institutions [3,4]. Recent clinical studies have demonstrated that elevated or persistently abnormal CEA and CA19-9 levels possess prognostic value in gastric cancer populations, including resectable cohorts treated with multimodal regimens [7] and postoperative series assessing dynamic marker trends [8]. Furthermore, marker elevation has been associated with advanced disease features and early treatment failure in real-world settings, supporting their use as adjuncts that may reflect differences in care pathways and disease phenotypes rather than serving as isolated predictors [9].

These observations are consistent with emerging evidence highlighting the role of tumour-associated inflammatory and molecular signalling pathways in gastric cancer progression and treatment response, as well as the contribution of targeted therapeutic strategies in biologically defined subgroups. Such findings further support the interpretation that biomarker profiles may reflect both underlying tumour biology and pathway-dependent clinical decision-making [10,11].

However, an important but often overlooked issue is that tumour marker testing may itself be dependent on the clinical pathway. The availability of CEA and CA19-9 results can cluster within specific diagnostic routes, such as emergency presentations compared to elective work-ups, or according to treatment intent, such as surgery-oriented versus non-surgical management. When biomarker testing is applied unevenly, analyses limited to patients who were tested may inadvertently select a subgroup defined by care processes rather than underlying biology. In real-world cohorts, it is therefore methodologically essential to report (i) the frequency of key marker availability, (ii) the clinical pathways associated with testing, and (iii) whether marker availability corresponds with staging completeness and treatment allocation.

Accordingly, this study investigates a consecutive, real-world cohort of patients with gastric cancer managed at a tertiary referral centre, with a focus on the structure of diagnostic documentation and its association with advanced disease and treatment selection. Specifically, the study (1) quantifies staging completeness, defining incomplete staging as the presence of Tx and/or Nx and/or Mx; (2) evaluates metastatic disease at diagnosis (M1) as a primary endpoint among patients with defined M status; and (3) characterises the availability patterns of CEA and CA19-9 and their associations with care pathways and clinical outcomes, without applying biomarker cut-offs. By centring the analysis on staging completeness and pathway-dependent biomarker testing, this work seeks to provide a pragmatic framework for interpreting advanced disease phenotypes and real-world treatment allocation in gastric cancer, while remaining consistent with contemporary staging principles and clinical guidance [3,4,5].

## 2. Materials and Methods

### 2.1. Study Design and Setting

This retrospective observational study was conducted at the Oncology and Surgical Departments of the Craiova County Emergency Hospital, a tertiary referral center serving southern Romania. The institutional oncology registry prospectively records newly diagnosed digestive malignancies and is routinely supplemented by electronic medical records.

For the present analysis, we focused on patients with gastric cancer diagnosed between January 2018 and December 2021. The aim of this third study in the research program was not to re-evaluate temporal trends, but to characterize diagnostic pathway architecture, staging completeness, and tumour marker testing patterns at presentation in a real-world cohort.

### 2.2. Study Population

All consecutive adult patients with histologically confirmed gastric carcinoma or adenocarcinoma diagnosed within the study window were eligible.

To reduce histological heterogeneity and ensure comparability of TNM-based analyses, the cohort was restricted to carcinoma/adenocarcinoma cases. Patients with non-epithelial gastric tumours or insufficient histopathological confirmation were excluded from the present analyses.

After applying these criteria, 375 patients constituted the analytical cohort for pathway-focused analyses. For analyses requiring a defined metastatic component (M0/M1), a subset of 360 patients with documented M status (M-defined subset) was used, with denominators reported accordingly.

### 2.3. Variables and Operational Definitions

Data were extracted into a structured dataset (Microsoft Excel 2020, Microsoft Corp., Redmond, WA, USA) and analyzed in IBM SPSS Statistics version 26.0 (IBM Corp., Armonk, NY, USA).

The following variables were evaluated:

• Demographic Variables
• Age at diagnosis (continuous; scaled per 10-year increase in regression models).
• Sex (male/female).
• Disease-Related Variables
• TNM components (8th edition).
• Metastatic disease at diagnosis (M1 vs. M0).
• Histological subtype (adenocarcinoma, carcinoma, discohesive type, mixed type).
• Endoscopic documentation (ED; yes/no) was defined as the presence of a narrative description of macroscopic disease extent/extension in the initial endoscopic or diagnostic records. ED was treated as a documentation/process variable and did not require a standardized reporting template or a complete TNM classification. Thus, ED reflected whether disease extent was explicitly described in the clinical record at presentation, rather than whether formal staging was complete.

Pathway Variables. Incomplete staging was defined a priori as the presence of at least one undefined TNM component (Tx and/or Nx and/or Mx). This variable was treated as a binary endpoint reflecting diagnostic completeness.

Both tumour markers available were defined as concurrent availability of numeric values for both CEA and CA19-9 at diagnosis. No predefined cut-offs were applied, as the objective was to evaluate testing patterns rather than diagnostic performance.

Treatment Variable. Surgery (any surgical intervention, yes/no), including both curative-intent and palliative procedures.

### 2.4. Statistical Analysis

Continuous variables are summarized as mean ± standard deviation (SD) or median with interquartile range (IQR), as appropriate. Categorical variables are summarized as counts and percentages. Univariable associations were evaluated using Pearson’s χ^2^ test for categorical variables. All statistical tests were two-sided with a significance threshold of α = 0.05.

Multivariable logistic regression models using the enter method were constructed to evaluate the following outcomes:

• Incomplete staging (Tx/Nx/Mx) as the primary pathway endpoint (restricted to the M-defined subset).
• Both tumour markers available (CEA + CA19-9) as a secondary pathway endpoint.
• Surgery (yes/no) as a complementary allocation model.
• Predictors entered into the models included metastatic status (M1 vs. M0), surgery (when not the outcome), ED (endoscopic documentation), histological subtype (with adenocarcinoma as reference), age (per 10-year increase), and sex.

Odds ratios (ORs) with 95% confidence intervals (CIs) were reported.

A complete-case approach was applied, and no statistical imputation was performed. Denominators were explicitly reported for each analysis to ensure transparency regarding missing data.

### 2.5. Ethics

The study was approved by the Ethics Committee of the University of Medicine and Pharmacy of Craiova (approval number 18593/13 April 2023) and conducted in accordance with the Declaration of Helsinki.

Given the retrospective design, exclusive use of routinely collected clinical data, and prior anonymization of identifiers, the requirement for individual informed consent was waived by the Ethics Committee.

## 3. Results

### 3.1. Study Cohort and Diagnostic Completeness

The final analytical cohort for this study comprised 375 patients with histologically confirmed gastric carcinoma/adenocarcinoma diagnosed between 2018 and 2021. In the full cohort (*n* = 375), incomplete TNM staging—defined a priori as the presence of Tx and/or Nx and/or Mx—was observed in 119 patients (31.7%), underscoring substantial heterogeneity in diagnostic documentation at presentation.

Among these 375 patients, 360 (96.0%) had a defined metastatic status (M0 or M1) and were included in analyses requiring M classification. Within this M-defined subset (*n* = 360), metastatic disease at diagnosis (M1) was identified in 185 patients (51.4%), indicating a high burden of advanced presentation in this real-world cohort.

Incomplete staging was not randomly distributed but showed marked variation across clinical characteristics and treatment pathways (Table 1).

### 3.2. Incomplete Staging and Clinical Pathways

Incomplete staging was strongly associated with indicators of advanced disease and non-surgical management. Patients with metastatic disease (M1) exhibited a significantly higher prevalence of incomplete staging compared with non-metastatic patients (50.3% vs. 6.3%, Pearson’s χ^2^ test, *p* < 0.001). Similarly, incomplete staging was markedly more frequent among patients who did not undergo surgery than among those treated surgically (54.7% vs. 10.7%, Pearson’s χ^2^ test, *p* < 0.001).

Endoscopic documentation (ED) showed a non-significant trend toward lower rates of incomplete staging (29.6% with ED vs. 40.5% without ED; Pearson’s χ^2^ test, *p* = 0.093), suggesting potential pathway-related differences in staging completeness, although this association did not reach statistical significance in univariable analysis.

Incomplete staging also varied across histologic subtypes within the carcinoma/adenocarcinoma spectrum (global Pearson’s χ^2^ test, *p* = 0.041), indicating that diagnostic completeness may differ according to tumour characteristics beyond simple stage classification.

### 3.3. Univariable Associations and Tumour Marker Testing Patterns

Univariable associations with incomplete staging and tumour marker availability are summarised in Table 2A,B. Multivariable results are presented in Section 3.5 (Table 3A).

Incomplete staging was strongly associated with metastatic presentation and non-surgical management (both *p* < 0.001). A modest but statistically significant global association was observed across histologic subtypes (*p* = 0.041), whereas the association with ED did not reach statistical significance (*p* = 0.093).

### 3.4. Testing Patterns for Tumour Markers (CEA and CA19-9)

Concurrent availability of both CEA and CA19-9 was documented in 138 of 360 patients (38.3%) in the M-defined subset (Table 2B).

Marker availability differed markedly according to treatment pathway. Among non-surgically managed patients, both markers were available in 49.2%, compared with 25.5% among surgically treated patients (*p* < 0.001).

ED was also associated with higher testing availability (40.9% vs. 20.3%, *p* = 0.002).

In contrast, no statistically significant difference in marker availability was observed between M0 and M1 patients (36.6% vs. 40.0%, *p* = 0.274).

Taken together, these findings indicate that biomarker testing appears to follow an organizational or pathway-dependent pattern rather than reflecting metastatic biology per se.

### 3.5. Multivariable Predictors of Incomplete Staging

Multivariable logistic regression (Table 3A) was performed in the M-defined subset (*n* = 360). After adjustment for age, sex, histology, ED, metastatic status, and surgery:

Metastatic disease (M1) remained strongly associated with incomplete staging (OR 10.98, 95% CI 5.25–22.94, *p* < 0.001).

Surgical management was independently associated with lower odds of incomplete staging (OR 0.14, 95% CI 0.07–0.27, *p* < 0.001).

ED showed an independent protective association (OR 0.31, 95% CI 0.14–0.66, *p* = 0.003).

Carcinoma histology (vs adenocarcinoma) was associated with higher odds of incomplete staging (OR 2.20, 95% CI 1.03–4.69, *p* = 0.042).

Age (per 10-year increase) and sex were not significantly associated with incomplete staging.

In this model, metastatic disease (M1) emerged as the strongest independent predictor of incomplete staging (OR 10.98, 95% CI 5.25–22.94; *p* < 0.001). Conversely, surgical treatment was independently associated with a substantially lower likelihood of incomplete staging (OR 0.14, 95% CI 0.07–0.27; *p* < 0.001). The presence of endoscopic documentation was also independently protective against incomplete staging (OR 0.31, 95% CI 0.14–0.66; *p* = 0.003).

After multivariable adjustment, incomplete staging remained independently associated with metastatic presentation, non-surgical management, ED, and carcinoma histology, while age and sex showed no significant associations.

**Figure 1:**
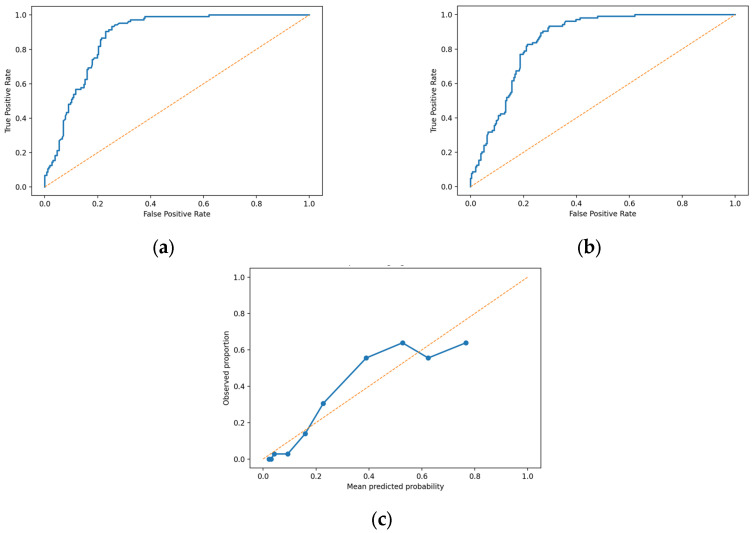
Discrimination and calibration of the multivariable model for incomplete staging (Tx/Nx/Mx) in the M-defined subset (*n* = 360): (**a**) Apparent ROC curve derived from the full dataset (AUC = 0.873); (**b**) Internal validation using 5-fold cross-validation (AUC = 0.855); (**c**) Calibration plot based on cross-validated predictions (10 quantile bins).

Model discrimination and internal calibration are summarised in Figure 1.

A complementary analysis was conducted with surgical management as the outcome. In a multivariable logistic regression model using surgical management (yes/no) as the dependent variable, metastatic presentation was associated with significantly lower odds of surgery (M1 vs. M0: OR 0.36, 95% CI 0.21–0.62; *p* < 0.001). Incomplete staging remained independently associated with non-surgical management (OR 0.15, 95% CI 0.08–0.29; *p* < 0.001), and ED also demonstrated an inverse association with surgery (OR 0.29, 95% CI 0.15–0.59; *p* < 0.001). This inverse association should be interpreted in a pathway context. Explicit documentation of disease extension was more frequently recorded in advanced or non-surgical trajectories in routine care, and therefore likely reflects clustering within specific diagnostic pathways rather than a causal effect of documentation on surgical decision-making.

Inclusion of concurrent biomarker availability (both CEA and CA19-9) in the model indicated that the presence of both markers continued to be associated with lower odds of surgery (OR 0.36, 95% CI 0.21–0.62; *p* < 0.001). The direction and magnitude of the effects for metastatic (M1) presentation and incomplete staging remained consistent.

These findings support the interpretation that both diagnostic completeness and biomarker testing reflect real-world pathway characteristics that are associated with non-surgical allocation, rather than representing isolated documentation artefacts.

### 3.6. Multivariable Predictors of Tumour Marker Availability

A second logistic regression model evaluated predictors of having both CEA and CA19-9 available (Table 3B).

After adjustment, surgical management was associated with significantly lower odds of marker availability (OR 0.38, 95% CI 0.23–0.63, *p* < 0.001).

ED was independently associated with higher testing availability (OR 3.01, 95% CI 1.56–5.82, *p* = 0.001).

Metastatic status, age, and sex were not significantly associated with testing availability.

These findings suggest that biomarker testing patterns are primarily determined by care pathway characteristics, rather than by metastatic burden or demographic variables.

**Figure 2:**
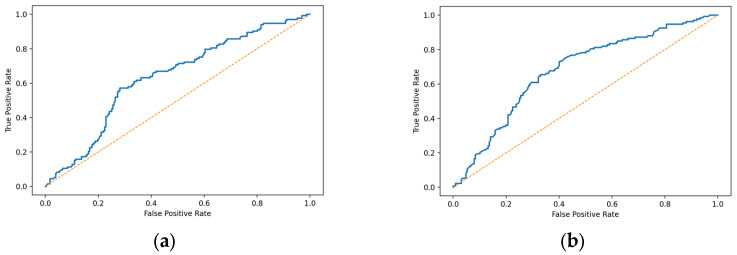
Receiver operating characteristic (ROC) curves for the multivariable model predicting availability of both tumour markers (CEA and CA19-9) in the M-defined subset (*n* = 360): (**a**) Apparent ROC curve (AUC = 0.680); (**b**) 5-fold cross-validated ROC curve (AUC = 0.633). The model included surgery status, ED, metastatic disease (M1), age (per 10-year increment), and sex. Discrimination was moderate and attenuated after internal validation, supporting the interpretation that tumour marker availability reflects pathway organization rather than a stable predictive rule.

Biomarker availability was independently associated with ED and non-surgical management, but not with metastatic status, age, or sex, thereby reinforcing the concept of pathway-driven testing. Discriminative performance of this model is shown in Figure 2.

## 4. Discussion

### 4.1. Principal Findings

In this real-world cohort of patients with histologically confirmed gastric carcinoma or adenocarcinoma, staging completeness at diagnosis exhibited substantial variability and was not randomly distributed across clinical pathways. Incomplete staging, defined as Tx, Nx, and/or Mx, demonstrated a strong association with metastatic presentation (M1) and non-surgical management, persisting after multivariable adjustment. In contrast, explicit documentation of disease extension was independently associated with a lower likelihood of incomplete staging and an increased probability of concurrent tumour marker availability.

Beyond the descriptive findings, the novelty of the present study lies in conceptualizing both staging completeness and tumour marker availability as pathway-dependent features of real-world diagnostic architecture. Rather than treating these variables as background noise or purely technical limitations, we analysed them as measurable indicators of how patients move through routine diagnostic and treatment pathways.

Current clinical guidelines emphasise comprehensive TNM staging as the foundation for treatment allocation in gastric cancer [3]. Nevertheless, real-world datasets frequently reveal substantial heterogeneity in stage documentation, especially among patients with advanced disease or those managed outside structured surgical pathways. Population-level analyses of large oncology datasets have shown that missing or partially defined variables are common and can be associated with survival and other clinical endpoints, supporting the notion that missingness may be structurally embedded rather than random [12,13]. These observations are consistent with interpreting staging incompleteness as a pathway-linked feature of routine care rather than an isolated documentation artifact.

These results extend this perspective by demonstrating that incomplete staging clusters specifically within metastatic and non-surgical trajectories. In these contexts, rapid triage to systemic or supportive care may compress the diagnostic workflow and limit full TNM characterisation. From a real-world evidence standpoint, recent reporting guidance emphasises that such data-generating mechanisms should be explicitly described and, where feasible, analytically addressed rather than implicitly ignored [13].

Tumour marker availability was also not uniformly distributed across the cohort. Both CEA and CA19-9 were more frequently available in patients managed without surgery and in those with explicit documentation of disease extension. Although serum tumour markers have demonstrated prognostic associations in gastric cancer cohorts [7], their testing patterns in routine practice may also reflect pathway-related organisational behaviours (e.g., differential work-up intensity, documentation practices, and care intent) rather than purely biological stratification. Contemporary real-world data frameworks highlight that availability of laboratory variables can be strongly influenced by clinical workflows, and thus should be interpreted in the context of fit-for-purpose data quality and pathway structure [14,15].

Taken together, these findings position staging completeness and tumour marker availability as measurable features of real-world diagnostic architecture. Explicit modelling of these variables complements prior literature focused primarily on stage distribution or survival outcomes and underscores the importance of pathway transparency when interpreting observational gastric cancer data [13,14,15].

### 4.2. Incomplete Staging as a Real-World Signal Rather than Random Missingness

In observational gastric cancer cohorts, incomplete staging is often regarded as a technical limitation inherent to retrospective data collection. However, evidence from contemporary oncology real-world datasets indicates that missing stage components and other key clinical variables are seldom random and may instead reflect underlying structural characteristics of care delivery and documentation [12,13]. Large-scale analyses have shown that patients with missing data can differ systematically from those with complete data, which has direct implications for inference if missingness is treated as ignorable by default [12].

These findings are consistent with our observations. In the present cohort, incomplete staging was independently and strongly associated with metastatic presentation and non-surgical management. This pattern supports the interpretation that incomplete TNM documentation may cluster within specific care pathways, particularly those characterised by rapid triage to systemic or palliative strategies. In such circumstances, clinical priorities may shift toward therapeutic decision-making rather than comprehensive anatomical staging, especially when metastatic disease is clinically apparent. Real-world evidence reporting guidance increasingly recommends that investigators describe these pathway-driven mechanisms explicitly—both to improve interpretability and to support cross-study comparability [13]

It is important to note that incomplete staging in this context does not inherently indicate diagnostic negligence or substandard care. Instead, it may reflect pragmatic decision-making under real-world constraints. Methodological work on oncology real-world data emphasises interpreting partially defined variables within the organisational and clinical contexts in which data are produced, including variation in documentation practices, diagnostic intensity, and care intent [14,15]. From this standpoint, incomplete staging emerges as a measurable characteristic of the diagnostic pathway itself.

The observed independent protective association between explicit documentation of disease extension and incomplete staging further substantiates the pathway interpretation. Systematic recording of disease extent is associated with improved staging completeness, indicating that documentation practices and diagnostic structures are critical to data integrity. Frameworks that operationalise “fit-for-purpose” real-world data quality similarly underline that completeness and traceability are not merely statistical properties, but reflections of process and workflow [15,16].

Importantly, ED should not be interpreted as a formal staging component. Rather, it captured the presence of descriptive documentation regarding disease extent in the initial record. Although conceptually related to staging completeness, ED and incomplete TNM staging were not equivalent constructs; accordingly, ED was included as a pathway-level documentation variable rather than a surrogate for the study endpoint.

Taken together, these findings suggest that staging completeness should be explicitly reported and analytically modelled in real-world gastric cancer research. Treating incomplete staging solely as a source of bias risks overlooking clinically meaningful pathway dynamics. Conversely, incorporating staging completeness as an endpoint enables a more nuanced interpretation of advanced disease phenotypes and treatment patterns, particularly in single-centre or registry-based cohorts [13,14,15,16].

### 4.3. Tumour Marker Testing as a Pathway-Driven Behaviour

In addition to evaluating staging completeness, this study investigated the patterns of serum tumour marker (CEA and CA19-9) availability at diagnosis. While these markers are associated with tumour burden and adverse clinicopathological features in gastric cancer cohorts [7], current guidelines do not recommend their use as independent decision tools for staging or treatment allocation [3]. Their clinical role remains primarily adjunctive, supporting contextual interpretation rather than substituting for anatomical classification.

Within this cohort, concurrent availability of both CEA and CA19-9 was significantly associated with non-surgical management and explicit documentation of disease extension. However, marker availability was not independently associated with metastatic status after multivariable adjustment. These findings indicate that tumour marker testing in routine practice may reflect organisational and pathway-related dynamics rather than solely biological stratification.

Recent research in real-world evidence has demonstrated that the availability of laboratory variables in retrospective datasets is strongly influenced by care processes, documentation practices, and workflow structures [14,15]. In oncology, testing intensity often varies according to treatment intent, perceived disease severity, or referral patterns. As a result, the presence of biomarker data may encode pathway information. Methodologically, this underscores the need to distinguish between biological and pathway signals when interpreting laboratory variables in observational cohorts.

The observed association between explicit documentation of disease extension and increased marker availability further supports this interpretation. Structured documentation is likely to correlate with a more comprehensive diagnostic work-up, including laboratory testing. Reporting guidelines for oncology real-world studies recommend transparent descriptions of data provenance and variable availability, especially when laboratory measures are included in analytic models [13]. Neglecting pathway-dependent testing may introduce selection mechanisms that distort effect estimates.

Importantly, these findings do not challenge the biological relevance of CEA and CA19-9. Instead, they highlight that, in real-world datasets, tumour markers may function at the intersection of biological and organisational factors. When analysed without predefined cut-offs and within a pathway framework, their availability and associations can clarify how diagnostic and treatment decisions are made in practice. Recognising this distinction is essential for drawing valid inferences from retrospective cohorts and supports a more nuanced interpretation of biomarker data in gastric cancer research.

### 4.4. Clinical and Methodological Implications

The findings of this study have both clinical and methodological implications. Clinically, staging completeness and tumour marker availability should be considered as indicators of care delivery in real-world settings, rather than merely as background variables. In gastric cancer, where treatment decisions depend on precise anatomical classification and multidisciplinary coordination [3], variability in staging documentation may affect both the interpretation of disease distribution and the assessment of eligibility for surgery or systemic therapy.

From a quality-of-care perspective, recent benchmark and standardization initiatives in gastric cancer surgery emphasize the importance of structured reporting and harmonized definitions to improve comparability across centres [17]. Inconsistent documentation practices can introduce unrecognized heterogeneity in observational cohorts, complicating inter-study comparisons. In gastric cancer specifically, recent international consensus initiatives have highlighted the need for harmonized reporting standards to improve reproducibility and facilitate benchmarking across centres [18].

Methodologically, the present analysis reinforces the need to conceptualize incomplete staging as a pathway-linked phenomenon rather than random data loss. Contemporary oncology real-world evidence (RWE) frameworks stress that data availability and completeness are intrinsically tied to clinical workflow, documentation culture, and care intent [13,14,15]. Regulatory and methodological discussions in oncology further underline that RWE studies must explicitly describe the data-generating process and potential structural biases to ensure interpretability and credibility [19]. By modelling incomplete staging and tumour marker availability as outcomes, our study operationalizes this principle within a single-centre gastric cancer cohort.

Moreover, explicit consideration of staging completeness may reduce implicit selection bias. If incomplete staging clusters within advanced or non-surgical pathways, excluding such cases without acknowledgement could distort the apparent distribution of disease severity or misrepresent treatment allocation patterns. Integrating completeness metrics into analytic models therefore enhances transparency and aligns with contemporary reporting guidance for observational health data [20].

Overall, these implications extend beyond gastric cancer. As oncology increasingly relies on real-world data for quality assessment, health services research, and hypothesis generation, pathway transparency and documentation integrity are becoming central methodological concerns. Integrating these considerations into analytic frameworks may improve comparability, reduce misinterpretation, and facilitate more accurate translation of observational findings into clinical insight.

### 4.5. Limitations

Several limitations must be considered when interpreting these findings.

First, the retrospective single-centre design limits external generalizability. While the cohort comprises consecutive real-world patients from a tertiary referral centre, documentation practices and pathway structures may vary across institutions and healthcare systems. Therefore, the observed associations between staging completeness, tumour marker availability, and treatment allocation should be interpreted within the specific organisational context in which the data were generated.

Second, incomplete staging, although analytically modelled as an outcome, is fundamentally a documentation-based construct. The absence of Tx, Nx, or Mx may reflect variability in recording practices rather than a lack of clinical evaluation. While this issue is central to the conceptual framework, residual misclassification remains possible. Similarly, tumour marker availability was analysed as a binary variable, without accounting for longitudinal testing patterns or treatment-response trajectories.

Third, the dataset did not include several potentially relevant variables that could influence staging and management pathways, such as standardised comorbidity indices (e.g., such as the Charlson score), performance status (e.g., ECOG), socioeconomic indicators, and detailed symptom-to-diagnosis intervals. These factors may confound associations between advanced presentation and diagnostic completeness, and their absence prevented more granular adjustment in multivariable models.

Fourth, although logistic regression models adjusted for key demographic and clinical covariates, the study was not powered for extensive interaction testing or causal inference. Therefore, the models should be regarded as exploratory and hypothesis-generating rather than definitive causal analyses.

Finally, survival outcomes were not the primary focus of this study. Although treatment allocation and metastatic presentation were examined, the prognostic implications of staging completeness and tumour marker testing were not assessed longitudinally. Future research integrating pathway architecture with time-to-event outcomes may further clarify the clinical impact of documentation variability.

Despite these limitations, the study’s strengths include the use of consecutive real-world cases, explicit operational definitions of incomplete staging and pathway endpoints, and multivariable modelling that incorporates documentation structure into the analytic framework. By highlighting staging completeness as a measurable pathway characteristic rather than treating it as background noise, this study provides a structured perspective for interpreting observational gastric cancer data.

## 5. Conclusions

In this real-world cohort of patients with gastric carcinoma and adenocarcinoma, staging completeness at diagnosis was not randomly distributed but strongly associated with metastatic presentation and non-surgical management. Incomplete TNM documentation clustered within advanced-disease pathways and remained independently linked to metastatic status after multivariable adjustment.

Tumour marker availability (CEA and CA19-9) similarly followed pathway-specific patterns, occurring more frequently in non-surgical and metastatic trajectories. These findings suggest that both staging completeness and biomarker testing function not merely as clinical descriptors but as structural indicators of diagnostic and therapeutic architecture in routine oncology practice.

By modelling incomplete staging as an analytic endpoint rather than treating it as background missingness, this study highlights the importance of pathway transparency in observational gastric cancer research. Explicit reporting and integration of documentation completeness may improve interpretability, reduce implicit bias, and enhance the methodological robustness of real-world datasets.

Future investigations integrating pathway metrics with longitudinal survival outcomes and multicentre validation are warranted to further clarify the clinical and quality-of-care implications of documentation variability in gastric cancer.

## Figures and Tables

**Table 1:** Baseline characteristics and diagnostic completeness in the carcinoma/adenocarcinoma cohort.

Characteristic	Value
Patients, *n*	375
Age, years (mean ± SD)	67.2 ± 12.0
Age, years (median [IQR])	69 [60–77]
Sex, male, *n* (%)	235 (62.7%)
Sex, female, *n* (%)	140 (37.3%)
ED documented, yes, *n* (%)	301 (80.3%)
ED documented, no, *n* (%)	74 (19.7%)
Surgery (any), yes, *n* (%)	196 (52.3%)
Surgery (any), no, *n* (%)	179 (47.7%)
Incomplete staging (Tx and/or Nx and/or Mx), *n* (%)	119/375 (31.7%)
M status defined (M0/M1), *n* (%)	360/375 (96.0%)
Metastatic disease among M-defined (M1), *n* (%)	185/360 (51.4%)
CEA available, *n* (%)	161/375 (42.9%)
CA19-9 available, *n* (%)	155/375(41.3%)
Both CEA + CA19-9 available, *n* (%)	138/375 (36.8%)
Histology (TIP): ADK, *n* (%)	223/375 (59.5%)
Histology (TIP): CARCINOM, *n* (%)	65/375 (17.3%)
Histology (TIP): DISCOEZIV, *n* (%)	56/375 (14.9%)
Histology (TIP): MIXT (incl. adenosquamous), *n* (%)	31/375 (8.3%)

Continuous variables are reported as mean ± SD and median [IQR]; categorical variables as *n* (%). Incomplete staging was defined a priori as Tx and/or Nx and/or Mx. “M-defined” indicates availability of M0/M1 classification.

**Table 2:** Univariable associations with incomplete staging and tumour marker testing availability. (**A**) Outcome: Incomplete staging (Tx/Nx/Mx). (**B**) Outcome: Both CEA + CA19-9 available.

(**A**)			
**Factor**	**Group**	**Incomplete,** ***n*/*N* (%)**	***p*** **-Value**
M status	M0	11/175 (6.3%)	**<0.001**
	M1	93/185 (50.3%)	
Surgery (any)	No	98/179 (54.7%)	**<0.001**
	Yes	21/196 (10.7%)	
ED documented	No	30/74 (40.5%)	0.093
	Yes	89/301 (29.6%)	
Histology (TIP2)	ADK	60/223 (26.9%)	**0.041**
	CARCINOM	28/65 (43.1%)	
	DISCOEZIV	19/56 (33.9%)	
	MIXT	12/31 (38.7%)	
(**B**)			
**Factor**	**Group**	**Both Markers Available, *n*/*N* (%)**	***p*** **-Value**
Surgery (any)	No	88/179 (49.2%)	**<0.001**
	Yes	50/196 (25.5%)	
ED documented	No	15/74 (20.3%)	**0.002**
	Yes	123/301 (40.9%)	
M status (M-defined subset)	M0	64/175 (36.6%)	0.274
	M1	74/185 (40.0%)	

Values in bold indicate statistically significant differences.

**Table 3:** Multivariable logistic regression models. (**A**) Primary model: predictors of incomplete staging. (**B**) Predictors of having both tumour markers available (CEA + CA19-9).

(**A**)				
**Predictor**	**OR**	**CI**		***p*** **-Value**
		**Low**	**High**	
Metastatic disease (M1 vs. M0)	10.98	5.25	22.94	**<0.001**
Surgery (yes vs. no)	0.14	0.07	0.27	**<0.001**
ED documented (yes vs. no)	0.31	0.14	0.66	**0.003**
TIP2: CARCINOM vs. ADK	2.20	1.03	4.69	0.042
TIP2: DISCOEZIV vs. ADK	1.41	0.68	2.93	0.353
TIP2: MIXT vs. ADK	1.67	0.72	3.87	0.232
Age (per 10 years)	0.91	0.74	1.12	0.379
Sex (male vs. female)	1.12	0.68	1.86	0.646
(**B**)				
Surgery (yes vs. no)	0.38	0.23	0.63	**<0.001**
ED documented (yes vs. no)	3.01	1.56	5.82	**0.001**
Metastatic disease (M1 vs. M0)	1.18	0.74	1.89	0.485
Age (per 10 years)	0.97	0.81	1.16	0.742
Sex (male vs. female)	1.09	0.67	1.77	0.731

Values in bold indicate statistically significant differences. (A) Outcome: Incomplete staging (Tx/Nx/Mx). Subset: M-defined (*n* = 360). (B) Outcome: Both markers available (yes/no). Subset: M-defined (*n* = 360).

## Data Availability

The authors declare that the data of this research are available from the corresponding authors upon reasonable request.
